# The Composition of Adipose-Derived Regenerative Cells Isolated from Lipoaspirate Using a Point of Care System Does Not Depend on the Subject’s Individual Age, Sex, Body Mass Index and Ethnicity

**DOI:** 10.3390/cells12010030

**Published:** 2022-12-21

**Authors:** Christoph Schmitz, Christopher Alt, Alon R. Azares, David A. Pearce, Tiffany R. Facile, John P. Furia, Nicola Maffulli, Claire Huang, Eckhard U. Alt

**Affiliations:** 1 Institute of Anatomy, Faculty of Medicine, LMU Munich, 80331 Munich, Germany; 2 Isar Klinikum Munich, 80331 Munich, Germany; 3 InGeneron, Inc., Houston, TX 77054, USA; 4 Molecular Cardiology Research Lab, Texas Heart Institute, Houston, TX 77030, USA; 5 Sanford Research, Sioux Falls, SD 57104, USA; 6 Sanford Health, Sioux Falls, SD 57117, USA; 7 Sanford School of Medicine, University of South Dakota, Sioux Falls, SD 57105, USA; 8 SUN Orthopedics of Evangelical Community Hospital, Lewisburg, PA 17837, USA; 9 Department of Musculoskeletal Disorders, Faculty of Medicine and Surgery, University of Salerno, 84084 Fisciano, Italy; 10 Centre for Sports and Exercise Medicine, Barts and The London School of Medicine and Dentistry, Mile End Hospital, Queen Mary University of London, London E1 2AD, UK; 11 School of Pharmacy and Bioengineering, Guy Hilton Research Centre, Keele University School of Medicine, Stoke on Trent ST4 7QB, UK; 12 Heart and Vascular Institute, Department of Medicine, Tulane University Health Science Center, New Orleans, LA 70112, USA

**Keywords:** adipose-derived regenerative cells, flow cytometry, microfragmented fat, NucleoCounter, regenerative medicine, stem cells, UA-ADRCs

## Abstract

Uncultured, unmodified, autologous, adipose-derived regenerative cells (UA-ADRCs) are a safe and effective treatment option for various musculoskeletal pathologies. However, it is unknown whether the composition of the final cell suspension systematically varies with the subject’s individual age, sex, body mass index and ethnicity. UA-ADRCs were isolated from lipoaspirate from *n* = 232 subjects undergoing elective lipoplasty using the Transpose RT system (InGeneron, Inc.; Houston, TX, USA). The UA-ADRCs were assessed for the number of nucleated cells, cell viability and the number of viable nucleated cells per gram of adipose tissue harvested. Cells from *n* = 37 subjects were further characterized using four-channel flow cytometry. The present study shows, for the first time, that key characteristics of UA-ADRCs can be independent of the subject’s age, sex, BMI and ethnicity. This result has important implications for the general applicability of UA-ADRCs in regeneration of musculoskeletal tissue. Future studies must determine whether the independence of key characteristics of UA-ADRCs of the subject’s individual age, sex, BMI and ethnicity only applies to the system used in the present study, or also to others of the more than 25 different experimental methods and commercially available systems used to isolate UA-ADRCs from lipoaspirate that have been described in the literature.

## 1. Introduction

Uncultured, unmodified, autologous, adipose-derived regenerative cells (UA-ADRCs) are a safe and effective treatment option for various musculoskeletal pathologies, including tendon injuries [1,2,3], bone defects [4], facet joint syndrome [5] and knee osteoarthritis [6]. Treatment with UA-ADRCs is a point of care procedure [1,7]. In the same surgical setting and within a very short time span (usually less than three hours), adipose tissue can be obtained by mini-liposuction, and the UA-ADRCs can be isolated and injected to the point in the body where they are needed [1,7,8].

Next to the term UA-ADRCs, the term stromal vascular fraction (SVF) is also often used in the literature (e.g., [9,10,11]). Both terms basically address the same uncultured, heterogeneous mixture of cells isolated from adipose tissue. The latter can be achieved with or without the use of enzymes (i.e., enzymatically or mechanically/non-enzymatically), with a significantly higher cell yield (number of nucleated cells per unit weight of adipose tissue or volume of lipoaspirate) with enzymatic methods than can be achieved with non-enzymatic/mechanical methods [12]. Cells that were isolated from adipose tissue in a manner that they are devoid of adipocytes but not of connective tissues (e.g., [13]) should not be regarded SVF/UA-ADRCs. In this respect, a recent systematic review argued that the term SVF is only suitable for enzymatic methods [14]. In any case, the SVF/UA-ADRCs must be differentiated from methods for generating so-called nanofat, described in the literature as mechanically emulsified fat tissue in a liquid form, ideally devoid of connective tissues but containing the cells of the SVF [15].

There are two main differences between the terms SVF and UA-ADRCs. First, SVF refers to the origin of the cells, whereas UA-ADRCs addresses both the origin of the cells and their use in medicine. Second, the term SVF does neither exclude the possibility that the cells were manipulated between isolation and use (e.g., by exposure to extracorporeal shock waves [16]), nor that they are used in an allogeneic manner [17,18,19]. For these reasons, only the term UA-ADRCs is used in the following text. 

The fact that treatment with UA-ADRCs is a point of care procedure raises the question of whether the composition of the final cell suspension of UA-ADRCs depends on the subject’s individual age, sex, body mass index (BMI) and ethnicity, which may cause interindividual differences in clinical results. Although more than 25 different experimental methods and commercially available systems used to isolate UA-ADRCs from lipoaspirate have been described in the literature [12,20,21,22,23,24,25,26], this important question has only been addressed in a very small number of studies so far. 

Specifically, one study [27] investigated lipoaspirate specimens from *n* = 52 female subjects (age range, 19–71 years; mean BMI, 18.59 ± 2.19 kg/m^2^ (mean ± standard deviation); all subjects white) and found a significant (*p* < 0.001), negative association between the subject’s age and the total number of nucleated cells per mL of adipose tissue. A limitation of this study [27] is the low mean BMI of the investigated subjects that is not representative of the mean BMI of subjects that were included in recent randomized controlled trials on treatment of partial-thickness rotator cuff tears [1] or knee osteoarthritis [28] with UA-ADRCs (the mean BMI of the subjects in [1] was 29.9 ± 5.7 kg/m^2^ (range, 20.8–42.3), and was 27.8 ± 3.9 kg/m^2^ (range, 19.6–34.9) in [28]).

Another study [29] investigated lipoaspirate specimens from *n* = 24 subjects (age range, 19–67 years; BMI range, 22.0–42.6 kg/m^2^; distributions of sex and ethnicity not provided). This study found no significant (*p* > 0.05) associations between (i) the subject’s age and the total number of nucleated cells per gram of adipose tissue, (ii) the subject’s age and the relative number (cells/cells) of CD45−/CD31−/CD34+ cells and (iii) the subject’s BMI and the total number of nucleated cells per gram of adipose tissue, but a significant (*p* = 0.006) negative association between the subject’s BMI and the relative number of CD45−/CD31−/CD34+ cells [29] (CD45−/CD31−/CD34+ cells were characterized as stromal fraction in [23], adipose progenitors in [25], SVF progenitor cells in [28] and mesenchymal stem/stromal cells in [29]). A limitation of the latter study [29] is the fact that the statistical analysis did not consider potential confounders (e.g., the subject’s BMI in analyses of associations between the subject’s age and numbers of cells, or the subject’s sex and ethnicity in all analyses).

It was therefore the aim of the present study to investigate the potential impact of the subject’s individual age, sex, BMI and ethnicity on the composition of the final cell suspension of UA-ADRCs using a sample of subjects that was more diverse than the sample of subjects investigated in [27], and better characterized than the sample of subjects investigated in [29]. Considering the limitations of the aforementioned studies [27,29], the null hypothesis of the present study was that the subject’s individual age, sex, BMI and ethnicity has no impact on the composition of the final cell suspension of UA-ADRCs.

This hypothesis was tested using the same enzymatic method to isolate UA-ADRCs from lipoaspirate that was used in [29]. Analyses were performed with a NucleoCounter NC-200 device (ChemoMetec Inc., Bohemia, NY, USA) and flow cytometry.

## 2. Materials and Methods 

The present study was approved by ADVARRA IRB (Columbia, MD, USA) (Protocol #200601001; approval issued on 16 October 2006; last continuing review approval issued on 24 August 2022). 

Lipoaspirate was harvested from the abdomen, bilateral flanks and/or medial thigh from *n* = 232 subjects undergoing elective lipoplasty according to standard procedures with informed consent. Lipoplasty was performed by a medical practitioner at The Aesthetic Center for Plastic Surgery (Houston, TX, USA) in the years 2017 to 2020. The Tumescent solution used for the liposuctions contained lidocaine (50 mL of 1% lidocaine in 3000 mL Tumescent solution) and epinephrine (3 mL of 1 mg/mL epinephrine in 3000 mL Tumescent solution). Lidocaine is known to be an effective local anesthetic in liposuction [30], with additional bacteriostatic effects [31,32] that are assumed to contribute to the extremely low incidence of infection seen in liposuction [30]. Epinephrine causes vasoconstriction, resulting in hemostasis and delayed absorption of the anesthetic agent in liposuction [30]). Liposuction was performed using long or short basket cannulas with diameters between 3 mm and 5 mm.

Lipoaspirate from all subjects was processed to isolate UA-ADRCs using the Transpose RT system (InGeneron) according to the manufacturer’s instructions. In very brief, lipoaspirate specimens were divided into four aliquots of approximately 25 mL each. Then, each aliquot was incubated together with Matrase Reagent (InGeneron) that contains collagenase I, collagenase II and a neutral protease for 30 min (the exact composition of the Matrase Reagent is proprietary). The latter was performed in the Transpose RT processing unit under agitation at 37 °C. The total procedure time was 70 min. The method is described in detail in [12]. 

Age, sex, BMI and ethnicity of the subjects as well as the tissue volume/weight used for four wash/processing tubes and the processing kit used (Transpose RT original or Transpose RT ultra; InGeneron) are summarized in Table 1.

The final cell suspension was analyzed for the following variables: V1, number of nucleated cells; V2, cell viability (%); V3, number of viable nucleated cells; V4, number of nucleated cells per gram tissue; and V5, number of viable nucleated cells per gram tissue. Cell counts and viability were determined using a NucleoCounter NC-200 device (ChemoMetec Inc., Bohemia, NY, USA) as described by the manufacturer’s protocol.

Furthermore, UA-ADRCs from *n* = 37 of the *n* = 232 subjects were investigated using flow cytometry. The latter was performed on fresh cells immediately after isolation. It was therefore not possible to collect all lipoaspirate specimens first, followed by selection of lipoaspirate specimens for characterizing UA-ADRCs using flow cytometry. To guarantee a sufficient number of cells for flow cytometry, the latter was preferentially performed on larger lipoaspirate specimens. No other selection criterion was applied. The *n* = 37 subjects whose lipoaspirate specimens were investigated using flow cytometry showed the following distributions of sex and ethnicity: female/male, 36/1; Caucasian/Hispanic/Black/Asian/African American/Arabic/Unknown, 26/3 5/2/0/1/0.

In order to lyse the last remaining erythrocytes in the cell suspension, the latter were incubated with 1X BD PharmLyse lysing solution (BD Biosciences, San Jose, CA, USA) prepared and used according to the manufacturer’s instructions prior to labeling freshly isolated UA-ADRCs with primary antibodies for flow cytometry. Afterwards, the cells were washed in FACS buffer (PBS containing 1% BSA and 0.05% sodium azide), followed by incubation for 30 min on ice with the conjugated, mouse-anti-human, primary antibodies listed in Table 2. 

Combinations of surface markers/primary antibodies and conjugates used for flow cytometry are summarized in Table 3.

After washing the cells twice with FACS buffer, flow cytometry was performed on a BD FACSCanto Flow Cytometer (BD Biosciences, Franklin Lakes, NJ, USA) using BD FACSDiva Software (BD Bioscience). On average 13,671 ± 3048 (mean ± standard deviation (SD)) (median, 13,308; range, 6222–21,588) live cell events were acquired. Analysis was performed using FlowJo Software (FlowJo, LLC, Ashland, OR, USA).

Mean, standard deviation (SD) and standard error of the mean (SEM) were calculated for all investigated variables.

Statistical analysis of the NucleoCounter results was performed using univariate ANOVA, with the subject’s sex, ethnicity and the kit used as fixed factors, and the subject’s age, subject’s BMI and the tissue volume/weight used for four wash/processing tubes as covariates. Post hoc analysis was performed using linear regression analysis and calculation of Pearson’s product moment correlation coefficient (r). 

The validity of the *n* = 37 lipoaspirate specimens that were investigated using flow cytometry as representative sample of the lipoaspirate specimens that were investigated using the NucleoCounter (ChemoMetec) was tested using the Kolmogorov-Smirnov test and Fisher’s exact test.

Statistical analysis of the flow cytometry results was performed using univariate ANOVA, with sex and ethnicity as fixed factors, and the tissue volume/weight used for four wash/processing tubes, the subject’s age, subject’s BMI, cell viability and the number of nucleated cells per gram tissue as covariates. Post hoc analysis was performed using linear regression analysis, calculation of Pearson’s product moment correlation coefficient and the Kruskal-Wallis test. 

All calculations were performed using IBM SPSS Statistics for Windows (Version 28.0.0.0; IBM Corp., Armonk, NY, USA) and GraphPad Prism (Version 9.4.1 for Windows; GraphPad Software, San Diego, CA, USA). In all analyses, an effect was considered statistically significant if its associated *p* value was smaller than 0.05.

## 3. Results

### 3.1. Results of the Investigations Using the NucleoCounter (ChemoMetec)

The results of the investigations using the NucleoCounter (ChemoMetec) are summarized in Table 4. Among these data, the most relevant were the mean cell viability (V2) (85.2% ± 4.78%; mean ± SD) and the mean number of nucleated cells per gram tissue (V4) (6.06 × 10^5^ ± 2.67 × 10^5^).

Univariate ANOVA (with sex, ethnicity and the kit used as fixed factors, and the subject’s age, subject’s BMI and the tissue volume/weight used for four wash/processing tubes as covariates) demonstrated a significant association between the subject’s BMI (B in Table 5) and the number of viable nucleated cells (V3 in Table 5) (*p* = 0.047), as well as between the subject’s BMI and the number of viable nucleated cells per gram tissue (V5 in Table 5) (*p* = 0.044). No other significant associations between the investigated variables V1-V5 and the subject’s age, subject’s sex, subject’s BMI, subject’s ethnicity, the tissue volume/weight used for four wash/processing tubes and the kit used were found (individual *p* values in Table 5). 

Post hoc linear regression analysis did not indicate significant associations between the subject’s BMI and the number of viable nucleated cells (r^2^ = 0.005; *p* = 0.275), as well as between the subject’s BMI and the number of viable nucleated cells per gram tissue (r^2^ = 0.006; *p* = 0.239). 

### 3.2. Validity of the Lipoaspirate Specimens Investigated Using Flow Cytometry As Representative Sample of the Lipoaspirate Specimens Investigated with the NucleoCounter (ChemoMetec)

**Figure 1:**
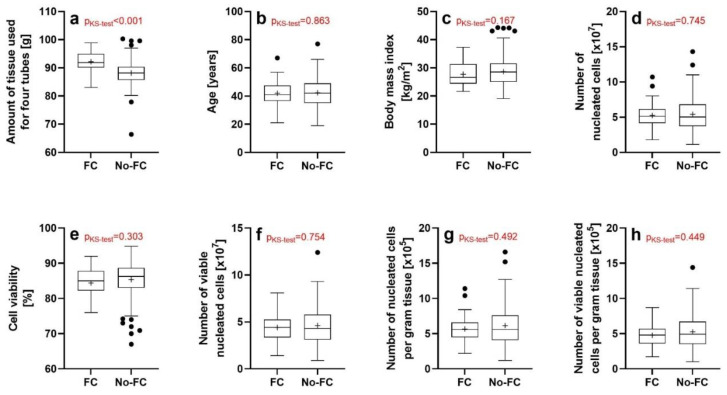
Tukey boxplots of (**a**) the amount of issue used, (**b**) subjects’ age, (**c**) subjects’ BMI, (**d**) number of viable nucleated cells, (**e**) cell viability, (**f**) number of viable nucleated cells, (**g**) number of nucleated cells per gram tissue and (**h**) number of viable nucleated cells per gram tissue of the lipoaspirate specimens that were investigated using flow cytometry (FC) and those lipoaspirate specimens that were not investigated using flow cytometry (No-FC). The results of the Kolmogorov-Smirnov test (KS) are given in red.

The Kolmogorov-Smirnov test showed a significant difference in the distributions of the amount of tissue used to isolate UA-ADRCs between the lipoaspirate specimens that were investigated with flow cytometry (hereafter: FC specimens) (on average 92.2 ± 3.4 g; mean ± SD) and those lipoaspirate specimens that were not investigated with flow cytometry (hereafter: No-FC specimens) (on average 88.2 ± 4.4 g) (*p* < 0.001) (Figure 1a). This was expected, as flow cytometry was preferentially performed on larger lipoaspirate specimens. No significant difference in the distributions of the subject’s age, subject’s BMI, the number of nucleated cells, cell viability, the number of viable nucleated cells, the number of nucleated cells per gram tissue and the number of viable nucleated cells per gram tissue were found using the Kolmogorov-Smirnov test between the FC specimens and the No-FC specimens (Figure 1b–h). 

Furthermore, Fisher’s exact test showed no significant difference in the distributions of the subject’s sex (*p* = 0.143) and subject’s ethnicity (*p* = 0.324) between the FC specimens and the No-FC specimens.

### 3.3. Results of the Flow Cytometry Investigations

Table 6 summarizes the combinations of surface markers/primary antibodies used for flow cytometry, the respective cell type as defined in the literature, the corresponding reference(s) to the literature, and the results of flow cytometry (relative numbers of cells (cells/cells) in per cent). 

Within the CD45− cell group (on average 58.0% ± 7.7%; mean ± SD) the highest mean relative number of cells was found for CD45−/CD31−/CD34+ cells (on average 32.8% ± 6.5%). The mean number of CD45−/CD31+/CD34+ cells was 15.3% ± 4.5%. 

Within the CD45+ cell group (on average 42.0% ± 7.7%; mean ± SD) the highest mean relative number of cells was found for CD45+/CD206+ cells (16.4% ± 4.3%).

Univariate ANOVA (with sex and ethnicity as fixed factors, and the tissue volume/weight used for four wash/processing tubes, the subject’s age, subject’s BMI, cell viability and the number of nucleated cells per gram tissue as covariates) demonstrated significant associations between: (i) the subject’s age and the relative numbers of CD45−/CD31−/CD34 cells (*p* = 0.049), CD45+/CD34+ cells (*p* = 0.045), CD31+ cells (*p* = 0.041), CD34+ cells (*p* = 0.008) and CD73+ cells (*p* = 0.017); (ii) the subject’s sex and the relative number of CD45−/CD73+/CD90+ cells (*p* = 0.002); (iii) the subject’s BMI and the relative number of CD34+ cells (*p* = 0.035); (iv) the subject’s ethnicity and the relative number of CD34+ cells (*p* = 0.030); and (v) the cell viability and the relative numbers of CD45−/CD31+ cells (*p* = 0.025), CD45−/CD31−/CD34− cells (*p* = 0.035) and CD45−/CD31−/CD34−/CD146+ cells (*p* = 0.037). No other significant associations between the investigated variables and the relative number of cells characterized by a certain combination of surface markers/primary antibodies were found (individual *p* values in Table 7). 

**Figure 2:**
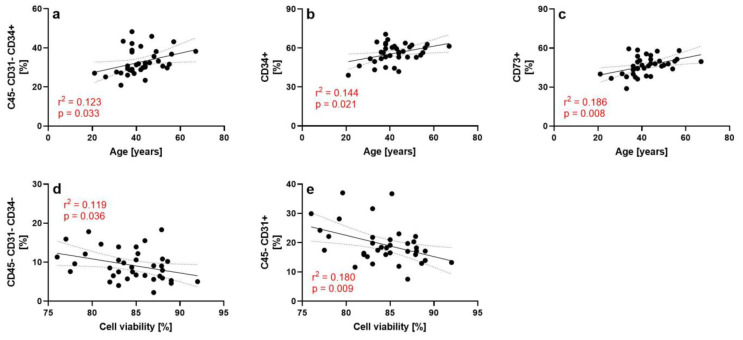
Individual relative numbers of cells of a certain cell type as a function of the subject’s age (**a**–**c**) and the cell viability (**d**,**e**) of the UA-ADRCs isolated from lipoaspirate using the Transpose RT system (InGeneron) of those subjects whose UA-ADRCs were analyzed using flow cytometry. The associations shown here are those that were significant in both univariate ANOVA (with fixed factors covariates) and post-hoc linear regression analysis. The results of linear regression analysis are given in red. Abbreviation: r, Pearson’s product moment correlation coefficient.

Post-hoc linear regression analysis showed significant associations between the subject’s age and the relative numbers of CD45−/CD31−/CD34+ cells (r^2^ = 0.123; *p* = 0.033), CD34+ cells (r^2^ = 0.114; *p* = 0.021) and CD73+ cells (r^2^ = 0.186; *p* = 0.008), as well as between the cell viability and the relative numbers of CD45−/CD31−/CD34− cells (r^2^ = 0.119; *p* = 0.036) and CD45−/CD31+ cells (r^2^ = 0.180; *p* = 0.009) (Figure 2). Post-hoc linear regression analysis did not show significant associations between: (i) the subject’s age and the relative number of CD45+/CD34+ cells (r^2^ = 0.129; *p* = 0.066); (i) the subject’s BMI and the relative number of CD34+ cells (r^2^ = 0.055; *p* = 0.163); and (iii) the cell viabiliy and the relative number of CD45−/CD31−/CD34−/CD146+ cells (r^2^ = 0.088; *p* = 0.075). Furthermore, the Kruskal-Wallis test did not show a significant association between the subject’s ethnicity and the relative number of CD34+ cells (*p* = 0.1196).

## 4. Discussion

This is the first study demonstrating that key characteristics of UA-ADRCs (number of nucleated cells, cell viability, the number of viable nucleated cells, the number of nucleated cells per gram tissue and the number of viable nucleated cells per gram tissue) can be independent of the subject’s age, sex, BMI and ethnicity. Accordingly, except for the significant, positive associations between the subject’s age and the relative numbers of cells shown in Figure 2a–c the null hypothesis of the present study was confirmed. This result has important implications for the general applicability of UA-ADRCs in regeneration of musculoskeletal tissue. The same applies to the characterization of UA-ADRCs using flow cytometry, although the presented data are limited by the fact that 36 of the 37 investigated lipoaspirate specimens were from women. Future studies must determine whether independence of key characteristics of UA-ADRCs of the subject’s age, sex, BMI and ethnicity only applies to the system used in the present study, or also to others of the more than 25 different experimental methods and commercially available systems used to isolate UA-ADRCs from lipoaspirate that have been described in the literature [12,20,21,22,23,24,25,26].

The mean values obtained using the NucleoCounter (ChemoMetec) in the present study were similar to corresponding data in other reports on the characterization of UA-ADRCs isolated from lipoaspirate using the Transpose RT system (InGeneron) [12,29]. Specifically, the mean number of nucleated cells per gram tissue was 6.06 × 10^5^ in the present study, 7.2 × 10^5^ in [12] and 5.9 × 10^5^ in [29]. Furthermore, the mean cell viability was 85.2% in the present study and 85.9% in [12]; cell viability was not reported in [29]. 

The latter study [29] also reported the results of the analysis of UA-ADRCs isolated from lipoaspirate of *n* = 24 subjects using flow cytometry (age range, 19–67 years; BMI range, 22.0–42.6 kg/m^2^; distributions of sex and ethnicity not provided). The mean relative number of CD45−/CD31−/CD34+ cells of 20.0% in [29] (compared to 32.8% in the present study) may be explained by the fact that frozen/thawed UA-ADRCs were investigated in [29], whereas fresh UA-ADRCs were investigated in the present study. Furthermore, the evidence of a significant (*p* = 0.006), negative association between the subject’s BMI and the relative number of CD45−/CD31−/CD34+ cells in [29], that was not found in the present study, may be explained by the facts that in [29] (i) a smaller sample was investigated than in the present study (*n* = 24 vs. *n* = 37), and (ii) the statistical analysis in [29] was a simple linear regression analysis, whereas in the present study univariate ANOVA with fixed factors and covariates was applied. On the other hand, the mean relative number of CD14+ cells reported in [29] (22.5%) was similar to the mean relative number of CD14+ cells found in the present study (18.9%). 

Collectively, these data support the reproducibility of results of characterizing UA-ADRCs isolated from lipoaspirate using the Transpose RT system (InGeneron).

The importance of a high cell viability of UA-ADRCs results from the fact that the injection of non-viable cells into tissue can lead to inflammatory reactions [37]. The International Federation for Adipose Therapeutics and Science (IFATS) has determined a cell viability of at least 70% as a minimum criterion for UA-ADRCs [10]. In the present study, this was achieved in 231 of the 232 (99.6%) investigated lipoaspirate specimens. In the literature, the mean cell viability was reported between 50% and 94% for enzymatic methods used to isolate UA-ADRCs from lipoaspirate [12], and between 64% [38] and 69% [39] for non-enzymatic/mechanical methods.

No explanation can be provided to date why the relative number of CD45−/CD31−/CD34+ cells increased with the subject’s age in the present study (Figure 2a). However, a pilot study showed that two samples of SVF collected from a healthy subject at age 72 years and again at age 84 years showed the same cell yield and SVF subpopulation composition, without change in the proliferation rate of adipose-derived stem cells (ADSCs) obtained by culturing SVF, as well as the capability of tri-lineage differentiation of both cell cultures [40]. Another pilot study showed that protein expression profiles of human umbilical vein endothelial cells (HUVECs) that were co-cultured under oxidative stress conditions with SVF from three healthy subjects aged 42, 45 and 47 years did not differ from protein expression profiles of HUVECs that were co-cultured under identical conditions with SVF from three healthy subjects aged 61 and 62 (two subjects) years [41].

In the present study, the relative numbers of CD45−/CD31−/CD34− cells and of CD45−/CD31+ cells (characterized as SVF nonprogenitors in [25]) increased with decreasing cell viability (Figure 2d,e). This finding implies that, with decreasing cell viability, those cell types that play an important role in regeneration of musculoskeletal tissue may be increasingly underrepresented in UA-ADRCs. This is in line with a finding of an earlier study in which a head-to-head comparison of four commercial systems used to isolate SVF/UA-ADRCs (Cytori StemSource 900/MB system (Lorem Cytori USA, Inc., San Diego, CA, USA) (hereafter: Cytori), PNC MultiStation (PNC Technologies Co., Ltd., Anyang, Republic of Korea) (hereafter: MultiStation), GID SVF-2 system (GID Bio, Inc., Louisville, CO, USA) (hereafter: GID SVF-2) and MediKhan Lipokit Platform (Medi Khan Inc., Seoul, Korea) (hereafter: Lipokit)) was performed [23]. Specifically, by investigating lipoaspirate specimens from 5 female subjects (age range, 25–37 years; BMI range, 21.8–28.3; the distribution of ethnicity not provided) the authors found that with decreasing average cell viability (Cytori (84.0%) > MultiStation (82.0%) > GID SVF-2 (69.3%) > Lipokit (50.3%) there was a trend towards decreasing relative numbers of CD45−/CD31−/CD34+ cells (Cytori (10.7%) > MultiStation (9.1%) > GID SVF-2 (8.9%) > Lipokit (7.2%) [23]. However, it would not be correct at this point to focus on just one cell type characterized by flow cytometry, as (i) no negative association between the cell viability and the relative number of CD45−/CD31−/CD34+ cells was found in the present study, and (ii) stem cells contained in UA-ADRCs are a heterogeneous population that cannot be fully characterized by flow cytometry [42]. Thus, full characterization of the stem cells (as well as of all other cell types) contained in UA-ADRCs will require large-scale single-cell transcriptomic sequencing, as recently performed for bone marrow aspirate concentrate (BMAC) [43]. Of note, corresponding data obtained for cultured ADSCs [44] are not suitable for drawing conclusions for UA-ADRCs, as stem cells contained in UA-ADRCs demonstrate rapid and marked changes in gene expression when subjected to standard tissue culture conditions [36,42].

In the present study, the CD45−/CD31−/CD34+ cells were the highest mean relative number of cells (on average 32.8% ± 6.5%) within the CD45− cell group. Adipose tissue derived progenitors and stem cells contained in UA-ADRCs play important roles in regeneration of musculoskeletal tissue (discussed in detail in, e.g., [1,2,3,4,5]). There is strong evidence indicating that the molecular and cellular mechanisms of action of UA-ADRCs in tendon regeneration are far beyond paracrine effects of the stem and progenitor cells contained in UA-ADRCs, as could be concluded from a number of recent studies [45,46,47,48,49]. Specifically, after the injection of cultured, autologous ADSCs into a rabbit Achilles tendon defect/repair model in vivo, the cells differentiated into tenocytes and integrated into the host tissue [50]. Furthermore, after the injection of cultured, autologous ADSCs into an experimentally induced tendon defect in horses in vivo, the ADSCs differentiated into cells that were integrated into new (tendon) tissue, with detection up to nine weeks post-treatment [51]. In addition, when seeding human, cultured ADSCs on a specific scaffold (Hyalonect meshes) in vitro, the ADSCs created a capillary network within the scaffold [52]. These results indicate that after injection of UA-ADRCs into a tendon defect, the cells may differentiate into other cell types that are necessary for tendon regeneration and integrate into the host tissue.

Within the CD45+ cell group, the CD45+/CD206+ cells (characterized as M2 macrophages in [25]) were the highest mean relative number of cells (16.4% ± 4.3%) in the present study. M2 macrophages are mainly involved in anti-inflammatory responses [53]. The tears of the rotator cuff were shown to be associated with synovial inflammation and increased expression of the pro-inflammatory markers interleukin-1β (IL-1β) and tumor necrosis factor-α (TNF-α) [54,55]. Furthermore, the exposure of cultured ADSCs with IL-1β and TNF-α resulted in decreased expression of the tenogenic transcription factor scleraxis [56]. The latter not only plays a pivotal role in promoting tenocyte proliferation and extracellular matrix (ECM) synthesis during embryonic tendon development, but is also involved in promoting the initial expansion of newly committed tenocytes and the production of ECM proteins in adult tendons [57]. This is in line with the finding that exposure of postnatal tendon cells with IL-1β resulted in reduced anabolic activity (leading to abnormal ECM deposition and organization) and increased catabolic activity (leading to proinflammatory cues and ECM degradation) [58]. In summary, the presence of M2 macrophages in UA-ADRCs may significantly contribute to tendon regeneration. Furthermore, the presence of M2 macrophages in UA-ADRCs may explain the very early treatment success observed after treating symptomatic, partial-thickness rotator cuff tears (sPTRCT) with UA-ADRCs [1,2], which cannot be explained by the formation of new tendon tissue.

The mean relative numbers of CD34+ cells and CD90+ cells found in the present study (56.0% and 56.6%) are in line with mean relative numbers of CD34+ cells and CD90+ cells reported in [36] (60% and 54.8%). This indicates that, at least when single surface markers are considered, different systems used to isolate UA-ADRCs from lipoaspirate can lead to very similar outcome. On the other hand, the mean relative number of CD105+ cells reported in the present study (24.1%) is much higher than the mean relative number of CD105+ cells reported in [36] (4.9%). This difference may arise from the fact that in [36] cells were isolated from lipoaspirate using 0.1% collagenase I. Collagenase I cleaves type I, type II and type III collagen but not type IV collagen [59], the main collagen of the basement membrane [60]. Type IV collagen is cleaved by neutral protease [61] which is contained in the Matrase Reagent (InGeneron) that was used in the present study. CD105 (endoglin) is found (among other cell types) on endothelial cells, endothelial progenitors [62,63] and ADSCs as defined by ISCT (CD45−/CD73+/CD90+/CD105+) [33]. The adhesion of these cell types to the basement membrane is achieved by α5β1 integrin [5,62,63]. Under physiological conditions, SPARC (secreted protein acidic and rich in cysteine; also known as osteonectin) can mobilize the aforementioned cell types through its effect on integrin α5β1, providing a functional basis for the regulation of the contribution of these cells to tissue and organ repair by SPARC [5,62,63]. The latter is synthesized by several cell types, including macrophages and infiltrating leukocytes [5]. Thus, SPARC may represent a key regulator in making endothelial progenitor cells and ADSCs as defined by ISCT a replacement source responsive to the signals of the surrounding tissue, and the neutral protease contained in the Matrase Reagent (InGeneron) may substitute the function of SPARC in isolation of UA-ADRCs from lipoaspirate.

The main limitation of the present study is the fact that 36 of the 37 lipoaspirate specimens investigated with flow cytometry were from women. This limitation is shared with other studies that reported the results of investigating UA-ADRCs with flow cytometry [23,25]. Therefore, we will verify the results of this experimental study performed on lipoaspirate specimens from subjects undergoing elective lipoplasty on lipoaspirate specimens that are currently collected in the framework of a large, randomized controlled trial (*n* = 246 subjects) on the treatment of sPTRCT with the injection of UA-ADRCs isolated from lipoaspirate using the Transpose RT system (InGeneron) vs. treatment with the injection of corticosteroid [64].

## 5. Conclusions

The present study shows, for the first time, that key characteristics of UA-ADRCs (number of nucleated cells, cell viability, the number of viable nucleated cells, the number of nucleated cells per gram tissue and the number of viable nucleated cells per gram tissue, as well as the results of flow cytometry) can be independent of the subject’s age, sex, BMI and ethnicity. This result has important implications for the general applicability of UA-ADRCs in the regeneration of musculoskeletal tissue. 

## Figures and Tables

**Table 1:** Age, sex, BMI and ethnicity of the *n* = 232 subjects whose lipoaspirate from elective lipoplasty was analyzed in the present study, as well as the amount of tissue used for four wash/processing tubes (Tissue) and the kit used (InGeneron).

Variable	Mean	SD	SEM	Minimum	Median	Maximum
**Age**	42.3	9.9	0.7	19	42	77
**BMI**	28.5	4.8	0.3	19.1	28.2	44.3
**Tissue [g]**	88.8	4.5	0.3	66.4	89.0	100.3
**Sex**	Female, *n* = 207; Male, *n* = 25					
**Ethnicity**	Caucasian, *n* = 153; Hispanic, *n* = 43; Black, *n* = 22; Asian, *n* = 7; African American, *n* = 3; Arabic, *n* = 2; Unknown, *n* = 2					
**Kit used**	Transpose RT original, *n* = 60; Transpose RT ultra, *n* = 172					

Abbreviations: SD, standard deviation; SEM, standard error of the mean; BMI, body mass index; g, grams.

**Table 2:** Primary antibodies used for flow cytometry in the present study.

CD	Clone	Isotype	Conjugate	Provider	Catalog #
CD3	OKT3	IgG2a, kappa	PE	eBioscience/Thermo Fisher	12-0037-42
CD4	RPA-T4	IgG1, kappa	FITC	eBioscience/Thermo Fisher	11-0049-42
CD14	61D3	IgG1, kappa	PE	eBioscience/Thermo Fisher	12-0149-42
CD16	CB16	IgG1, kappa	FITC	eBioscience/Thermo Fisher	11-0168-42
CD19	HIB19	IgG1, kappa	APC	eBioscience/Thermo Fisher	17-0199-42
CD25	BC96	IgG1, kappa	PE	eBioscience/Thermo Fisher	12-0259-42
CD31	WM59	IgG1, kappa	PE	eBioscience/Thermo Fisher	12-0319-42
CD33	WM53	IgG1, kappa	FITC	eBioscience/Thermo Fisher	11-0338-42
CD34	581	IgG1, kappa	PE-Cy	BD Pharmingen/BD Biosciences	555823
CD45	HI30	IgG1	PerCP	Thermo Fisher	MHCD4531
CD45	HI30	IgG1, kappa	PerCP-eFluor71m	eBioscience/fisher scientific	50-245-943
CD73	AD2	IgG1, kappa	APC	eBioscience/Thermo Fisher	17-0739-42
CD90	5E10	IgG1, kappa	PE	eBioscience/Thermo Fisher	12-0909-42
CD105	MEM-226	IgG2a	FITC	Thermo Fisher	MA1-19594
CD117	YB5.B8	IgG1, kappa	PE	eBioscience/Thermo Fisher	12-1179-42
CD127	eBioRDR5	IgG1, kappa	APC	eBioscience/Thermo Fisher	12-1179-42
CD144	16B1	IgG1	Alexa Fluor 488	eBioscience/Thermo Fisher	53-1449-42
CD146	P1H12	IgG1, kappa	FITC	eBioscience/Thermo Fisher	11-1469-42
CD206	19.2	IgG1, kappa	APC	eBioscience/Thermo Fisher	17-2069-42

Manufacturers and providers: eBioscience/Thermo Fisher (Waltham, MA, USA); BD Pharmingen/BD Biosciences (San Jose, CA, USA). Abbreviations: CD, cluster of differentiation; PE, phycoerythrin; FITC, fluorescein isothiocyanate; APC, allophycocyanin; PE-Cy, R-phycoerythrin-cyanine complex; PerCP; peridinin-chlorophyll-protein complex.

**Table 3:** Combinations of surface markers/primary antibodies (M) and conjugates (C) used for flow cytometry in the present study.

Flow Cytometry Tube 1		Flow Cytometry Tube 2		Flow Cytometry Tube 3		Flow Cytometry Tube 4	
M	C	M	C	M	C	M	C
CD45	PerCP	CD45	PerCP	CD45	PerCP	CD45	PerCP
CD73	APC	CD4	FITC	CD34	PE-Cy	CD14	PE
CD90	PE	CD25	PE	CD105	FITC	CD16	FITC
CD105	FITC	CD127	APC	CD117	PE	CD206	APC
**Flow Cytometry Tube 5**		**Flow Cytometry Tube 6**		**Flow Cytometry Tube 7**			
**M**	**C**	**M**	**C**	**M**	**C**		
CD45	PerCP	CD45	PerCP	CD45	PerCP		
CD3	PE	CD31	PE	CD31	PE		
CD19	APC	CD34	PE-Cy	CD34	PE-Cy		
CD33	FITC	CD146	FITC	CD144	Alexa Fluor 488		

**Table 4:** Mean, standard deviation (SD), standard error of the mean (SEM), minimum, median and maximum of the number of nucleated cells (V1), cell viability (V2), number of viable nucleated cells (V3), number of nucleated cells per gram tissue (V4) and number of viable nucleated cells per gram tissue (V5) of the UA-ADRCs isolated from lipoaspirate using the Transpose RT system (InGeneron) of *n* = 232 subjects undergoing elective lipoplasty.

Variable	Mean	SD	SEM	Minimum	Median	Maximum
V1 [×10^7^]	5.38	2.33	0.15	1.1	5.0	14.3
V2 [%]	85.2	4.78	0.31	67.0	86.0	94.8
V3 [×10^7^]	4.59	1.98	0.13	0.9	4.4	12.4
V4 [×10^5^/g]	6.06	2.67	0.18	1.2	5.6	16.6
V5 [×10^5^/g]	5.18	2.28	0.15	1.0	4.9	14.4

**Table 5:** Results (*p* values) of the statistical analysis (univariate ANOVA with fixed factors and covariates) of the data shown in Table 4. *p* values < 0.05 are given boldface.

	A	S	B	E	T	K	K × S	K × E	S × E	K × S × E
V1	0.077	0.141	0.075	0.139	0.155	0.707	0.582	0.857	0.979	0.154
V2	0.669	0.978	0.294	0.416	0.312	0.840	0.154	0.432	0.454	0.662
V3	0.085	0.125	**0.047**	0.085	0.216	0.719	0.655	0.807	0.963	0.146
V4	0.073	0.110	0.071	0.112	0.854	0.769	0.565	0.833	0.979	0.124
V5	0.081	0.097	**0.044**	0.066	0.738	0.773	0.638	0.773	0.961	0.117

Abbreviations: A, age; S, sex; B, body mass index; E, ethnicity; T, tissue volume/weight used for four wash/ processing tubes; K, kit used; V1, number of nucleated cells; V2, cell viability; V3, number of viable nucleated cells; V4, number of nucleated cells per gram tissue; V5, number of viable nucleated cells per gram tissue.

**Table 6:** Combination of surface markers/primary antibodies used for flow cytometry, the respective cell type as defined in the literature, the corresponding reference to the literature, and the results of flow cytometry (mean, standard deviation, standard error of the mean, minimum, median and maximum of the relative numbers of cells (cells/cells in per cent) that were positive for the tested combination of surface markers/primary antibodies).

Surface Markers	Cell Type	Reference	n	Mean	SD	SEM	Min	Med	Max
CD45-	CD45- cell group		37	58.0	7.7	1.3	42.1	57.6	72.1
CD45- CD73+ CD90+	(ADSCs)	[33]	37	32.5	8.7	1.4	20.4	30.0	61.4
CD45- CD73+ CD90+ CD105+	ADSCs	[33]	37	2.4	1.4	0.2	0.2	2.0	6.7
CD45- CD31+	Endothelial cells	[25]	37	19.3	6.5	1.1	7.5	17.4	37.0
CD45- CD31+ CD34+	Endothelial progenitors	[25]	37	15.3	4.5	0.7	7.3	14.4	28.1
CD45- CD31+ CD34+ CD146+	Pericytes	[25]	37	13.2	4.0	0.7	3.8	13.2	24.9
CD45- CD31-	CD45- CD31- group	[25]	37	36.5	5.5	0.9	28.3	35.6	51.8
CD45- CD31- CD34+	Stromal fraction Adipose progenitors SVF progenitor cells Mesenchymal stem/stromal cells	[23] [25] [28] [29]	37	32.8	6.5	1.1	20.9	30.9	48.3
CD45- CD31- CD34+ CD146+	Pericyte progenitors	[25]	37	0.7	0.4	0.1	0.1	0.5	2.1
CD45- CD31- CD34-	SVF nonprogenitors	[25]	37	9.2	4.0	0.7	2.2	8.7	18.3
CD45- CD31- CD34- CD146+	Pericytes	[25]	37	6.5	4.5	0.7	0.6	5.4	16.0
CD45+	CD45+ cell group		37	42.0	7.7	1.3	27.9	42.4	57.9
CD45+ CD34+	Leukocyte progenitors	[25]	27	1.7	1.4	0.3	0.6	1.4	7.8
CD45+ CD206+	M2 macrophages	[25]	37	16.4	4.3	0.7	8.1	16.0	26.1
CD45+ CD4+ CD25-	Naïve T cells	[34]	20	4.0	2.0	0.4	1.4	3.8	9.0
CD45+ CD4+ CD25+	Regulatory T cells	[35]	20	4.3	1.9	0.4	0.7	4.8	8.6
CD14+		[29]	37	18.9	5.8	1.0	8.1	18.4	31.8
CD31+		[36]	37	49.6	5.8	0.9	39.1	50.8	58.8
CD34+		[36]	37	56.0	7.3	1.2	39.0	56.7	70.6
CD73+		[36]	37	46.5	7.1	1.2	28.8	46.8	59.4
CD90+		[36]	37	56.6	8.0	1.3	40.6	57.8	74.8
CD105+		[36]	37	24.1	6.1	1.0	2.3	24.4	38.1

Abbreviations: n, number of lipoaspirate specimens investigated; SD, standard deviation; SEM, standard error of the mean; Min, minimum value; Med, median value; Max, maximum value.

**Table 7:** Results (*p* values) of the statistical analysis (univariate ANOVA with fixed factors and covariates) of the data shown in Table 7. *p* values < 0.05 are given boldface.

Surface Markers	A	S	BMI	E	T	V2	V4
CD45−	0.973	0.632	0.411	0.411	0.847	0.139	0.141
CD45− CD73+ CD90+	0.100	**0.002**	0.373	0.402	0.483	0.617	0.060
CD45− CD73+ CD90+ CD105+	0.858	0.547	0.769	0.762	0.464	0.156	0.295
CD45− CD31+	0.306	0.494	0.469	0.648	0.659	**0.025**	0.343
CD45− CD31 + CD34+	0.390	0.836	0.198	0.962	0.702	0.089	0.232
CD45− CD31+ CD34+ CD146+	0.625	0.930	0.455	0.998	0.755	0.458	0.686
CD45− CD31-	0.157	0.914	0.918	0.638	0.508	0.656	0.386
CD45− CD31− CD34+	**0.049**	0.908	0.462	0.397	0.742	0.186	0.819
CD45− CD31− CD34+ CD146+	0.976	0.724	0.456	0.688	0.706	0.731	0.441
CD45− CD31− CD34-	0.099	0.719	0.507	0.339	0.852	**0.035**	0.129
CD45− CD31− CD34− CD146+	0.145	0.680	0.114	0.179	0.578	**0.037**	0.545
CD45+	0.969	0.625	0.406	0.437	0.859	0.139	0.148
CD45+ CD34+	**0.045**	--*	0.186	0.600	0.073	0.521	0.976
CD45+ CD206+	0.747	0.483	0.409	0.781	0.657	0.313	0.488
CD45+ CD4+ CD25-	0.390	--*	0.188	0.487	0.697	0.182	0.956
CD45+ CD4+ CD25+	0.139	--*	0.572	0.278	0.645	0.108	0.501
CD14+	0.919	0.794	0.181	0.292	0.709	0.440	0.952
CD31+	**0.041**	0.931	0.757	0.372	0.454	0.362	0.327
CD34+	**0.008**	0.826	**0.035**	**0.030**	0.820	0.274	0.478
CD73+	**0.017**	0.671	0.553	0.073	0.934	0.293	0.617
CD90+	0.273	0.245	0.351	0.368	0.671	0.941	0.356
CD105+	0.590	0.903	0.601	0.610	0.854	0.664	0.267

Abbreviations: A, age; S, sex; BMI, body mass index; E, ethnicity; T, tissue volume/weight used for four wash/processing tubes; V2, cell viability; V4, number of nucleated cells per gram tissue; *, no data available for male subjects.

## Data Availability

All raw data (including the files that were obtained from the BD FACSDiva Software (BD Bioscience)) are available from the corresponding author on reasonable request.
